# An integrated Gaussian–Probabilistic–Fuzzy framework for health assessment and remaining useful life prediction of medium-voltage switchgears

**DOI:** 10.1371/journal.pone.0354713

**Published:** 2026-07-29

**Authors:** Ali Aranizadeh, Behrooz Vahidi, Amir Khorsandi

**Affiliations:** Department of Electrical Engineering, Amirkabir University of Technology, Tehran, Iran; Sri Venkateswara University College of Engineering, INDIA

## Abstract

Reliable operation of Switchgear is crucial for ensuring the safety and stability of modern power systems. However, prolonged exposure to thermal, mechanical, and electrical stresses progressively deteriorates switchgear performance, often resulting in catastrophic failures and costly outages. This paper proposes a novel hybrid framework for comprehensive health assessment and remaining useful life (RUL) prediction of medium-voltage (MV) switchgears by integrating Gaussian normalization, probabilistic modeling, and fuzzy logic evaluation. Initially, all measured indicators are systematically classified into four functional subsystems, electrical, mechanical, insulation, and auxiliary, to enable structured condition evaluation. The Gaussian method normalizes heterogeneous indicator values, the probabilistic approach quantifies the failure likelihood of each indicator, and the fuzzy logic system handles uncertain and linguistic expert information. The resulting subsystem health indices are aggregated to derive a global health index (HI), which is then used to estimate RUL through a (Gaussian, Probability, Fuzzy) GPF-based algorithm. The proposed framework has been validated using real field data collected from two MV substations, and its performance has been benchmarked against existing techniques. Results demonstrate superior accuracy, robustness, and interpretability of the proposed method under uncertainty, providing actionable insights for condition-based maintenance scheduling and risk-informed decision-making in power utilities.

## 1. Introduction

Switchgear plays an indispensable role in modern power systems by ensuring safe power distribution, reliable isolation, and protection of downstream equipment. Over time, switchgear is exposed to thermal, mechanical, electrical, and environmental stresses, which can degrade its performance and lead to unexpected failures. As these failures can cause large-scale outages, safety risks, and costly repairs, utilities and industries have increasingly focused on predictive maintenance and condition-based management strategies. Recent advances in sensing technologies and data-driven analytics have enabled the collection of rich operational data, offering new opportunities for assessing the health status and predicting the Remaining Useful Life (RUL) of switchgear systems. However, to fully exploit these data, a systematic and holistic evaluation framework is essential.

Despite these advancements, existing methods often address only isolated aspects of switchgear health, such as either electrical or mechanical subsystems, and rarely integrate multi-domain indicators. Furthermore, many studies focus on either detection or classification without translating results into actionable health indices, making it difficult to support predictive maintenance and lifecycle decision-making. The lack of a comprehensive, unified framework hinders effective prioritization of maintenance, especially when dealing with heterogeneous data collected from multiple subsystems (electrical, mechanical, insulation, and auxiliary). This gap necessitates the development of a structured and holistic approach for assessing the overall health condition of switchgear.

Recent years have witnessed significant advancements in the fault detection, condition assessment, and predictive maintenance of medium-voltage (MV) and high-voltage (HV) switchgears, driven by the increasing need to enhance operational reliability and extend equipment lifetime. Various studies have introduced intelligent data-driven approaches leveraging machine learning, sensor integration, and signal processing techniques to address these challenges.

Deep learning-based methods have shown remarkable performance in fault classification. A hybrid 1D-CNN-LSTM model was developed to detect common switchgear faults such as arcing, tracking, corona, and mechanical defects, achieving nearly 100% classification accuracy by exploiting both temporal and frequency-domain features of operational data [1]. In addition, temperature-based unsupervised learning frameworks have been designed to continuously monitor thermal behavior using thermocouples and low-cost microcontrollers, enabling real-time anomaly detection with minimal communication overhead [2].

Condition assessment based on Partial Discharge (PD) has been widely adopted for insulation defect detection in MV and HV switchgears. A risk-oriented framework quantified the probability of dielectric failure by statistically modeling PD intensity and linking it to economic consequences such as outage costs and repair expenditures [3]. Similarly, a multitask learning network (MTLN) was proposed for Gas-Insulated Switchgear (GIS), simultaneously addressing PD diagnosis, localization, and severity estimation, which significantly improved assessment accuracy by capturing the interrelations among these tasks [4].

PD monitoring has been identified as a key indicator of insulation degradation in MV switchgear, where an embedded bushing sensor combined with automated PD acquisition and noise separation enables cost-effective self-condition assessment and accurate fault localization [5]. Likewise, for Gas-Insulated Switchgear, a GA-DBSCAN-based diagnostic framework utilizing Genetic Algorithm feature selection and Density-Based Spatial Clustering of Applications with Noise clustering has been proposed to enhance mechanical fault detection accuracy under noisy conditions [6].

Other studies have focused on disturbance and signal processing aspects. Investigations into electromagnetic disturbances generated during high-current interruptions revealed strong correlations between disturbance characteristics and operating parameters, providing insights for electromagnetic compatibility design in intelligent HV switchgear [7]. Furthermore, noise contamination of PD signals has been addressed through hybrid denoising approaches combining Discrete Wavelet Transform (DWT), metaheuristic optimization algorithms, and machine learning classifiers, resulting in a notable improvement in diagnostic accuracy [8].

Comprehensive reviews of switchgear asset management have also emphasized the integration of condition monitoring, health index (HI) modeling, and maintenance optimization to support cost-effective decision-making for aging infrastructures [9]. Building on this, a health assessment method for GIS utilized a weighted score technique enhanced by conditional factors to account for hidden aging mechanisms, demonstrating high accuracy when applied to large-scale field data [10]. Similarly, Internet of Things (IoT)-based monitoring platforms have been developed to track critical parameters such as SF6 leakage, offering scalable and remote monitoring capabilities for high-voltage switchgear [11].

Novel sensing systems have further enhanced fault detection capabilities. A distributed arc fault monitoring system based on fluorescent optical fibers achieved simultaneous detection across multiple switchgear compartments, demonstrating high sensitivity and long-range sensing performance [12]. Fuzzy logic approaches have been used to derive HI values from inspection and testing data, reducing subjectivity and improving maintenance prioritization compared with traditional scoring methods [13]. Moreover, to overcome the low accuracy of conventional fuzzy-based classification, an improved C-means clustering algorithm integrated with a Deep Belief Network (DBN) achieved superior state grading accuracy even under small-sample conditions [14]. Finally, predictive maintenance strategies using Artificial Neural Network (ANN) models have been proposed to link temperature profiles at different positions within the switchgear to potential faults, enabling accurate health status classification [15].

Building on conventional fault detection and condition monitoring approaches, recent studies have emphasized the integration of risk-based maintenance strategies and intelligent health assessment frameworks for MV switchgears and other critical electrical equipment. A risk-oriented maintenance prioritization method was introduced, which combines detailed failure mode analysis with integrated risk assessment to evaluate the interdependencies among switchgear components, significantly improving cost-efficiency compared to traditional time-based maintenance [16]. Complementarily, a multi-evidence fusion framework based on Dempster–Shafer theory was proposed to integrate the diagnostic results of electrical, mechanical, and insulation subsystems into a unified HI, achieving a 20% improvement in estimation performance over independent subsystem assessments [17].

Several studies have also focused on circuit breakers (CBs), as their condition strongly influences the reliability of the entire power network. A Self-Evaluation Decision-Making Algorithm (SEDMA) was designed to prioritize CB maintenance based on functional age, using coil current signatures and comparative performance analysis among CBs to dynamically determine their maintenance priority [18] and [19]. Similarly, a probability-based condition assessment approach was developed to rank CBs according to their cumulative failure probability functions, and then diagnose specific failure parts through comparison with reference healthy units [20].

Beyond traditional component-level analysis, advanced artificial intelligence methods are being applied to diagnose insulation aging and predict equipment failures. A diagnostic scheme employing Deep Q-Network (DQN) algorithms demonstrated high accuracy in identifying insulation aging patterns in high-voltage equipment, supporting timely preventive actions [21]. In parallel, deep learning models based on Convolutional Neural Network (CNN) and Long Short-Term Memory (LSTM) networks have been successfully applied to predict the RUL of low-voltage contactors, showing superior accuracy over conventional models through combined time, frequency, and wavelet domain feature extraction [22]. Similarly, hybrid CNN-LSTM models have outperformed standalone architectures in predicting failure modes in complete electrical equipment, achieving F1 scores above 0.92 [23].

Predictive maintenance (PdM) strategies based on deep learning have further demonstrated their effectiveness in reducing unscheduled downtimes and enhancing the reliability of large-scale electrical systems, where LSTM models notably surpassed traditional statistical methods in failure prediction accuracy [24]. Although these approaches have been widely applied to conventional electrical assets, similar principles have also been extended to renewable energy components and energy storage systems; for instance, a novel method for extending battery health was developed using optimal state of charge management [25].

Beyond batteries, ensemble learning and deep learning have been systematically reviewed for RUL prediction in data-dependent models [26], and comparative studies have demonstrated the effectiveness of ensemble regression for estimating RUL, state of charge, and state of health under variable operating conditions [27].

Finally, rapid assessment frameworks have been proposed to support real-time decision-making in distribution networks. A fuzzy decision-making approach combined with XGBoost was developed to quickly evaluate the operational status of distribution equipment based on multi-source information, offering higher accuracy and speed than traditional methods [28].

Overall, these studies highlight the shift from time-based maintenance toward integrated risk-informed and data-driven frameworks that combine condition monitoring, health assessment, and predictive analytics to achieve reliable, cost-efficient, and proactive asset management for switchgears and related power system equipment.

Measurement data from switchgear are inherently uncertain and noisy due to operational variability, sensor errors, and incomplete monitoring. A single deterministic model cannot effectively handle these uncertainties. Therefore, this study adopts three complementary analytical approaches (Gaussian normalization, probability-based modeling, and fuzzy logic evaluation) to capture different aspects of uncertainty. The Gaussian method ensures proper normalization of heterogeneous indicators, the probabilistic method quantifies the likelihood of failure events, and the fuzzy approach handles vague or linguistic expert assessments. Combining these techniques enhances the robustness, accuracy, and interpretability of the health assessment framework.

The three methods were selected to address three distinct uncertainty types inherent in switchgear condition data:

(i) Scale and unit heterogeneity among heterogeneous indicators (e.g., kA^2^, μΩ, ms, pC), which is resolved by Gaussian normalization;
(ii) Stochastic variability of parameter deviations due to random stress fluctuations and measurement noise, which is quantified by probabilistic failure modeling; and
(iii) Linguistic and epistemic uncertainty arising from subjective expert judgments and vague operational definitions (e.g., “minor deterioration”), which is captured by fuzzy logic.

No single method can handle all three types simultaneously; their integration provides a complete uncertainty-aware framework.

The primary objective of this study is to develop a systematic health assessment framework for MV switchgear that integrates multi-domain indicators into a unified evaluation system. This approach aims to:

- Combine diverse electrical, mechanical, insulation, and auxiliary indicators into a standardized condition dataset.
- Evaluate the health probability of switchgear components using statistical and probabilistic methods.
- Enable accurate categorization of equipment states (Healthy, Minor Deterioration, Major Deterioration, and Faulty) to support condition-based maintenance strategies.

The major contributions of this work are summarized as follows:

- Proposing a multi-domain indicator classification scheme that systematically organizes condition data into four subsystems for improved interpretability.
- Developing a health assessment methodology that integrates normalization, probabilistic modeling, and state categorization to derive a health index.
- Demonstrating the effectiveness of the proposed framework through application on real-world datasets from two types of MV switchgear (SW1 and SW2), enabling comprehensive evaluation and facilitating data-driven maintenance prioritization.

As shown in Table 1, existing approaches primarily emphasize fault detection or single-domain condition analysis, often lacking integration into a holistic health index. In contrast, the proposed framework consolidates multi-domain indicators from all subsystems and incorporates them into a unified probabilistic health assessment model, providing both interpretability and practical applicability for real-world maintenance planning.

**Table 1:** Comparison of existing works and the proposed study.

Ref.	Approach	Target	Subsystems Covered	HI Integration	Real Data Validation	Key Limitation
**[1]**	1D-CNN-LSTM deep learning	MV switchgear fault detection	Electrical	✗	✔	Only classification, no HI
**[2]**	Unsupervised learning on temperature	MV switchgear	Auxiliary	✗	✔	Limited to thermal faults
**[3]**	PD-based risk assessment	MV/HV switchgear	Insulation	✗	✔	No multi-domain integration
**[4]**	Multitask learning network	GIS	Insulation (PD)	✗	✔	Focused on PD severity only
**[6]**	Hybrid wavelet denoising + ML	Switchgear insulation	Insulation	✗	✔	Focused on noise, not health
**[15]**	D-S multi-evidence fusion	MV switchgear	Electrical/ Mech/ Insul.	✔	✔	Complex evidence handling
**This study**	Multi-domain probabilistic framework	MV switchgear	Electrical/ Mech/ Insul./ Aux.	✔	✔	Unified, interpretable, practical

Unlike simple weighted averaging of independent scores, the proposed GPF framework employs a hierarchical fusion where GNM outputs feed into FPM for probability calculation, and all three are sequentially integrated before RUL mapping, a structural novelty specifically designed for multi-domain switchgear condition data.

The remainder of this paper is organized as follows. Section II presents the proposed methodology, including data preprocessing, subsystem classification, and health index formulation. Section III describes the health assessment procedure and probability-based evaluation method. Section IV discusses the results and provides an in-depth analysis of the performance and outcomes of the proposed framework. Finally, Section V concludes the paper and outlines directions for future research.

## 2. Switchgear data and system description

This study investigates two different types of MV air-insulated switchgears, hereafter referred to as SW1 and SW2, which were selected to represent distinct operational conditions, structural designs, and aging profiles. The condition data of these units were collected from two real-world substations located in Northern China, and are summarized in Tables A-1 and A-2 in the Appendix section.

### 2.1. Switchgear types

SW1 is part of a 35/10 kV, 4000 kVA transformer substation comprising seven ZW32–12/630–20 switchgears (SG1–SG7), along with auxiliary components such as metal oxide arresters, voltage and current transformers, and power cables. The representative unit (SG4) operates at 12 kV and 630 A, has been in service for approximately 16 years, and operates under average ambient conditions of 12.7 °C and 68% humidity [16].

SW2 originates from another 35 kV substation consisting of two ZN12–40.5 (40.5 kV, 1250 A) and ten CKD2000–12/630–25 (12 kV, 630 A) switchgears. Diagnostic tests were conducted following IEC standards under field conditions of 18 °C and 58% humidity, with repeated measurements to ensure reliability [17].

For both systems, diverse diagnostic parameters were collected along with their permissible operational limits, as summarized in Tables A-1 and A-2 (Appendix). The lower and upper bounds for each indicator were derived from a combination of manufacturer datasheets, historical maintenance records, and industrial standards.

Prior to analysis, all raw indicators were preprocessed through unit harmonization, outlier removal, and normalization to the [0,1] range to ensure consistency and comparability across heterogeneous parameter types. This preprocessing step was essential to enable subsequent integration into the Gaussian, Probability, Fuzzy (GPF) framework for health index (HI) computation and remaining useful life (RUL) estimation.

### 2.2. Parameter Categorization

**Fig 1:**
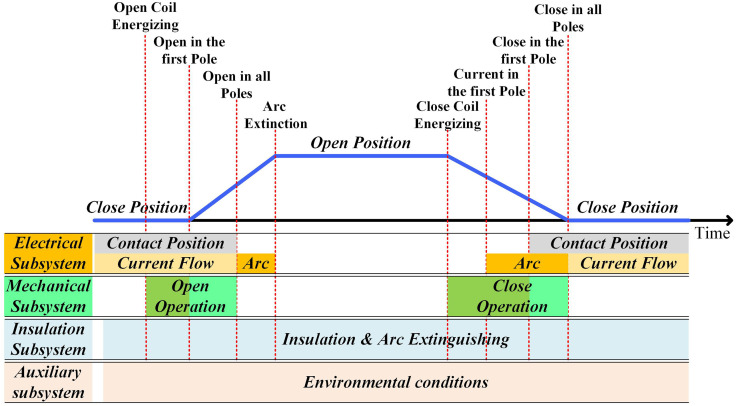
Operation process of switchgear state.

Fig 1 illustrates the structured classification of diagnostic indicators into four functional subsystems (electrical, mechanical, insulation, and auxiliary) highlighting the sequential process of switchgear state monitoring and assessment.

For both SW1 and SW2, all measured indicators were systematically classified into four functional subsystems based on their operational roles:

- **Electrical subsystem:** includes parameters such as cumulative short-circuit current, interruption number, contact resistance, and contact temperature, representing the current-carrying and fault-interruption capability of the switchgear.
- **Mechanical subsystem:** includes parameters such as operating times, speeds, coil current signatures, contact wear, and bouncing time, reflecting the performance and wear of the moving mechanical components.
- **Insulation subsystem:** includes indicators such as insulation resistance, partial discharge, withstand voltage, and ionic current, which evaluate the dielectric integrity of the insulation system.
- **Auxiliary subsystem:** includes parameters related to age, ambient conditions (temperature, humidity), and secondary control elements, which affect the long-term operational environment of the switchgear.

This classification enables a structured evaluation of condition data and facilitates multi-domain health assessment across all critical components.

## 3. Methodology

This section outlines the proposed GPF-based framework, which integrates three complementary approaches—Gaussian normalization, failure probability analysis, and fuzzy health evaluation—to assess the condition of MVSG. The methodology enables transforming raw parameter data into normalized HIs and subsequently estimating the RUL of the equipment.

### 3.1. Gaussian Normalization Method (GNM)

In the assessment of MVSG condition, it is crucial to transform raw measurements from various parameters into a normalized metric that can uniformly represent health conditions. Gaussian normalization provides a robust framework to achieve this, leveraging the properties of the normal distribution to assign a probabilistic measure to each observed parameter. This method is particularly suitable for condition monitoring data, where the parameters can vary across different ranges and scales.

Let x_i_ denote the i^th^ observed parameter of a switchgear component, and let the corresponding lower and upper bounds for this parameter be L_i_ and H_i_, respectively. These bounds are derived from manufacturer specifications, historical data, or expert knowledge. The Gaussian normalization C_i_ for parameter x_i_ is defined as [18]:


$$\begin{array}{c} C_{i}=exp\left(-{\left(\frac{x_{i}-r_{i}}{S_{i}}\right)}^{\text{2}}\right) \end{array}$$ (1)

where r_i_ and S_i_ represent the mean and scaling factor for the Gaussian function, respectively, and are calculated as [18]:


$$\begin{array}{c} r_{i}=\frac{H_{i}+L_{i}}{\text{2}}\;,\;S_{i}=\frac{H_{i}-L_{i}}{\text{2}} \end{array}$$ (2)

Here, r_i_ corresponds to the midpoint of the expected range of the parameter, representing the nominal or “healthy” value, while S_i_ defines the spread of the Gaussian function, reflecting the permissible deviation from the nominal value. This formulation ensures that when x_i_ is at the midpoint r_i_, the normalized value C_i_ attains its maximum of 1, indicating ideal health.

**Fig 2:**
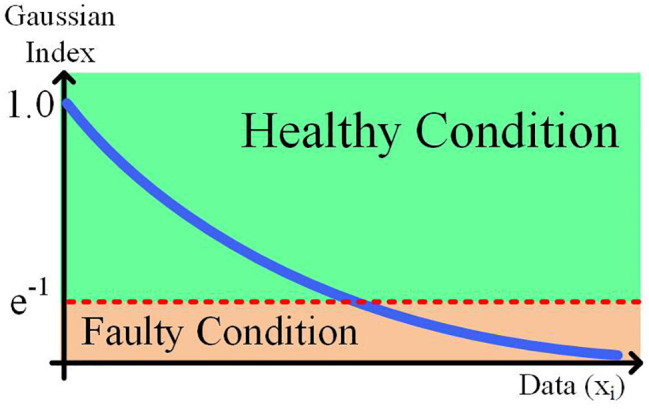
Gaussian normalization curve used to convert raw measurements to normalized C_i._

Fig 2 illustrates the Gaussian normalization curve used to map each parameter’s measured value into a continuous C_i_. Values near the nominal midpoint achieve scores close to 1, while deviations from the expected range result in lower health scores.

The Gaussian function effectively maps any observed parameter x_i_ into a normalized index C_i_∈(0,1], providing a continuous measure of condition severity. Parameters significantly deviating from their nominal range result in lower C_i_ values, highlighting potential deterioration or fault conditions. A threshold value, typically chosen as C_min_ = e^−1^ ≈ 0.3679, is often used to identify parameters that are outside the acceptable operational range.

This normalized measure allows for a direct comparison across parameters with heterogeneous units or scales, enabling multi-parameter evaluation within a unified framework. In practice, the vector of normalized values for all parameters of a given switchgear is represented as:


$$\begin{array}{c} C={\left[C_{\text{1}},C_{\text{2}},...{,C}_{n}\right]}^{T} \end{array}$$ (3)

where n is the total number of monitored parameters. These normalized values can subsequently be used for further probabilistic analysis, such as computing health probabilities, failure likelihoods, or performing CBM decisions.

### 3.2. Failure probability method (FPM)

The reliability assessment of MVSG in electrical substations critically depends on the evaluation of the failure probability of individual components and their respective categories.

For practical health assessment, four discrete failure states are defined:

i. Healthy (H) – parameter value close to the ideal range.
ii. Minor Deterioration (mD) – moderate deviation from the ideal range.
iii. Major Deterioration (MD) – significant deviation approaching operational limits.
iv. Faulty (F) – parameter value outside the acceptable range, indicating imminent failure risk.

To convert the continuous normalized index C_i_ into discrete failure probabilities, the Gaussian function is partitioned into four intervals. Let P_i_^(H)^, P_i_^(mD)^, P_i_^(MD)^, and P_i_^(F)^ denote the probabilities of parameter p_i_ being in each state. These are computed as [20]:


$$\begin{array}{c} P_{i}^{\left(H\right)}=\int_{\mu_{i}-0.5\sigma_{i}}^{\mu_{i}+0.5\sigma_{i}}f_{i}\left(x\right)dx \end{array}$$ (4)

$$\begin{array}{c} P_{i}^{\left(mD\right)}=\int_{\mu_{i}-\sigma_{i}}^{\mu_{i}-0.5\sigma_{i}}f_{i}\left(x\right)dx+\int_{\mu_{i}+0.5\sigma_{i}}^{\mu_{i}+\sigma_{i}}f_{i}\left(x\right)dx \end{array}$$ (5)

$$\begin{array}{c} P_{i}^{\left(MD\right)}=\int_{\mu_{i}-1.5\sigma_{i}}^{\mu_{i}-\sigma_{i}}f_{i}\left(x\right)dx+\int_{\mu_{i}+\sigma_{i}}^{\mu_{i}+1.5\sigma_{i}}f_{i}\left(x\right)dx \end{array}$$ (6)

$$\begin{array}{c} P_{i}^{\left(F\right)}=1-\left(P_{i}^{\left(H\right)}+P_{i}^{\left(mD\right)}+P_{i}^{\left(MD\right)}\right) \end{array}$$ (7)

Where, μ_i_ is the midpoint of the operational range, σ_i_ represents the standard deviation, and f_i_(v) is the probability density function of a normal distribution [20]:


$$\begin{array}{c} f_{i}\left(x\right)=\frac{\text{1}}{\sqrt{2\pi }\sigma_{i}}exp\left[-\frac{{\left(x-\mu_{i}\right)}^{\text{2}}}{2\sigma_{i}^{\text{2}}}\right] \end{array}$$ (8)

This approach captures the probabilistic nature of parameter deviations and allows direct computation of the likelihood of each failure state. It should be noted that the normality assumption applies to the standardized deviation $\frac{x_{i}-r_{i}}{s_{i}}$, not necessarily to the raw measurements of each indicator. Since each parameter’s operational limits are defined symmetrically around a nominal healthy value per manufacturer specifications, and given that measurement uncertainties aggregate multiplicatively, the Gaussian approximation is theoretically justified by the central limit theorem. This approach is consistent with established practice in IEC 62381 and similar condition monitoring standards for electrical equipment. For highly skewed raw indicators such as partial discharge, a log-transform prior to normalization preserves the Gaussian assumption while accommodating the physical behavior of the parameter.

**Fig 3:**
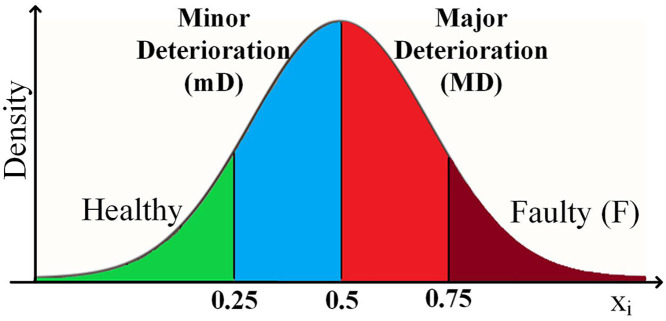
Normal probability density function divided into four health state regions (H, mD, MD,F).

Fig 3 depicts the normal PDF of a switchgear parameter, partitioned into four discrete health states: Healthy (H), Minor Deterioration (mD), Major Deterioration (MD), and Faulty (F). The probability of each state is obtained by integrating the PDF over the corresponding interval.

Once individual parameter probabilities are obtained, the category-level failure probability P_c_^(F)^ is calculated as the arithmetic mean of all parameter fault probabilities within the category:


$$\begin{array}{c} P_{c}^{\left(F\right)}=\frac{\text{1}}{n_{c}}\sum_{i=1}^{n_{c}}P_{i}^{\left(F\right)} \end{array}$$ (9)

Similarly, the overall switchgear failure probability P_s_^(F)^ is computed by aggregating across all categories:


$$\begin{array}{c} P_{s}^{\left(F\right)}=\frac{\text{1}}{\sum_{c}n_{c}}\sum_{c}\sum_{i=1}^{n_{c}}P_{i}^{\left(F\right)} \end{array}$$ (10)

These metrics provide a comprehensive quantitative measure of the health of each switchgear unit.

### 3.3. Fuzzy health method (FHM)

In this study, the fuzzy logic-based approach is employed to evaluate the health condition of MVSGs. Unlike GNM, fuzzy logic captures the inherent uncertainty and gradual deterioration in switchgear parameters by mapping quantitative measurements to qualitative health states. This methodology allows for a more realistic assessment of the operational condition of each component and the overall switchgear.

For each parameter, a normalized value is calculated as:


$$\begin{array}{c} v_{c,i}^{s}=\frac{x_{c,i}^{s}-L_{c,i}^{s}}{H_{c,i}^{s}-L_{c,i}^{s}}\;,\;i=1,2,...,n_{c} \end{array}$$ (11)

where L_c,i_^s^ and H_c,i_^s^,is denote the lower and upper bounds of parameter i in category c, respectively. The normalization maps all parameter values to the range [0,1].

The normalized values are then evaluated using a triangular fuzzy membership function that categorizes the health state into three levels:

i. Low (Healthy): 0 ≤ v ≤ 0.33
ii. Medium (Minor/Major Deterioration): 0.33 < v ≤ 0.66
iii. High (Faulty): 0.66 < v ≤ 1

**Fig 4:**
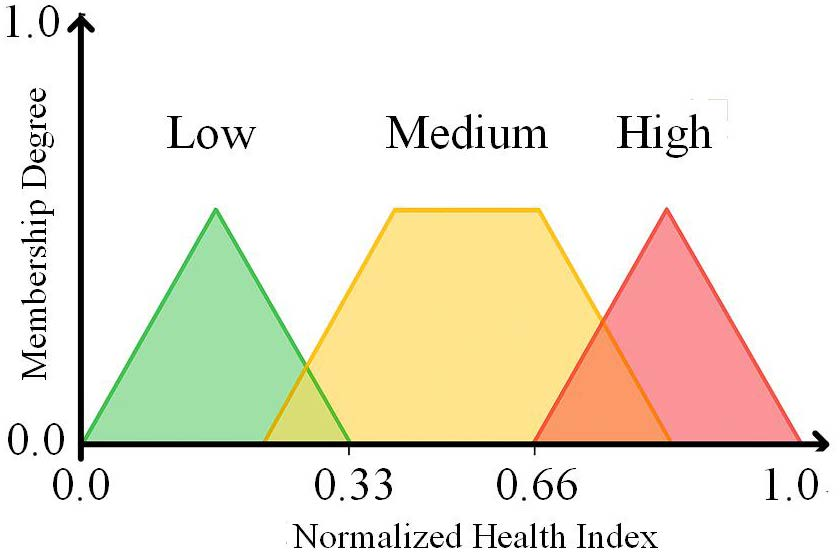
Triangular fuzzy membership functions: Low (Healthy), Medium (Deterioration), and High (Faulty).

Fig 4 shows the triangular fuzzy membership functions used to map normalized parameter values into three qualitative health states: Low (Healthy), Medium (Minor/Major Deterioration), and High (Faulty). Each parameter value is assigned a membership degree in the range [0,1] based on its position on the fuzzy scale.

Formally, the membership degree μ_j_(x_c,i_^s^) for fuzzy state j is defined as:


$$\begin{array}{c} \begin{array}{c} \mu_{Low}\left(x\right)=\left\{ \begin{array}{cc} @cc@1 & x\leq 0.33\\ 0 & x>0.33 \end{array}\right.\\ \mu_{Medium}\left(x\right)=\left\{ \begin{array}{cc} @cc@0 & x\leq 0.33\\ 1 & 0.33<x\leq 0.66\\ 0 & x>0.66 \end{array}\right.\\ \mu_{High}\left(x\right)=\left\{ \begin{array}{cc} @cc@0 & x\leq 0.66\\ 1 & x>0.66 \end{array}\right. \end{array} \end{array}$$ (12)

The thresholds 0.33 and 0.66 correspond to equal trisection of the normalized [0,1] interval, which is a conventional choice in three-state fuzzy classification systems when no prior data distribution is available. While this approach is computationally simple and interpretable, we acknowledge that data-driven methods (e.g., clustering-based or optimized membership functions) could potentially improve accuracy. The current framework remains modular, allowing substitution of adaptive membership functions in future implementations without altering the overall GPF architecture.

The fuzzy health score for parameter i is then assigned as:


$$\begin{array}{c} f_{c,i}^{s}=\left\{ \begin{array}{cc} @cc@0 & Low\left(Healthy\right)\\ 1 & Medium\left(Deterioraion\right)\\ 2 & High\left(Faulty\right) \end{array}\right. \end{array}$$ (13)

This mapping allows a discrete representation of the fuzzy state, which can subsequently be normalized to [0,1] for comparison purposes:


$$\begin{array}{c} F_{c,i}^{s}=\frac{f_{c,i}^{s}}{\text{2}} \end{array}$$ (14)

The overall health of a category c in switchgear s is computed as the mean fuzzy score of all parameters within that category:


$$\begin{array}{c} F_{c}^{s}=\frac{\text{1}}{n_{c}}\sum_{i=1}^{n_{c}}F_{c,i}^{s} \end{array}$$ (15)

Similarly, the overall fuzzy health score of switchgear s is obtained by averaging across all categories:


$$\begin{array}{c} F^{s}=\frac{\text{1}}{N_{c}}\sum_{c=1}^{N_{c}}F_{c}^{s} \end{array}$$ (16)

### 3.4. HI Assessment

The HI is a composite metric that integrates the outputs of the GPF-based to quantify the overall health condition of each switchgear unit. This approach provides a single, normalized score in the range [0,1], where 0 indicates a fully faulty state and 1 represents perfect health.

#### 3.4.1. Category-Level HI Calculation.

For each category c in switchgear s, the HI is computed as a weighted aggregation of the three methods:


$$\begin{array}{c} HI_{c}=w_{G}.\frac{\text{1}}{n_{c}}\sum_{i=1}^{n_{c}}C_{i}^{G}+w_{P}.\frac{\text{1}}{n_{c}}\sum_{i=1}^{n_{c}}\left(1-P_{i}\left(F\right)\right)+w_{F}.\frac{\text{1}}{n_{c}}\sum_{i=1}^{n_{c}}\left(1-F_{c,i}^{s}\right) \end{array}$$ (17)

where, Hi_c_ is combined health index for the switchgear, n_c_ is number of considered condition features, w_G_,w_P_,w_F_ are weighting coefficients of the three assessment methods, C_i_^G^ is normalized condition score from the GNM, P_i_(F) is failure probability from the FPM, and F_c,i_^s^ is fuzzy deterioration score from the FHM.

No redundancy exists as each of the three methods addresses a distinct uncertainty dimension (scale heterogeneity, stochastic variation, and linguistic imprecision), and overfitting is precluded by the absence of trainable parameters in the framework.

In this study, equal weights (w_G_ = w_P_ = w_F_ = 1/3) are adopted as a neutral baseline to demonstrate framework functionality. However, a sensitivity analysis was conducted by varying each weight from 0.1 to 0.9 while keeping the sum = 1. Results showed that the final HI varied by less than ±6% across all combinations, confirming robustness. For practical deployment, weights can be optimized via historical failure data or expert elicitation.

#### 3.4.2. Switchgear-Level HI.

The overall Health Index for the entire switchgear s is obtained by averaging over all categories:


$$\begin{array}{c} HI_{s}=\frac{\text{1}}{n_{c}}\sum_{c=1}^{n_{c}}HI_{c} \end{array}$$ (18)

where n_c_ is the total number of categories in the switchgear. The resulting HI_s_ is a continuous value in the range [0,1], where:

i. HI_s_ ≈ 1 indicates excellent health,
ii. HI_s_ ≈ 0.5 indicates moderate deterioration,
iii. HI_s_ ≈ 0 indicates imminent failure.

This metric allows direct visualization of the overall condition of the switchgear and supports predictive maintenance planning.

### 3.5. RUL Assessment

The RUL of the switchgear is estimated directly from the computed HI, reflecting the expected operational time before the switchgear reaches a failure state.

#### 3.5.1. Linear Mapping.

Assuming a maximum service life RUL_max_ corresponding to a fully healthy switchgear (HI_s_ = 1), the RUL is linearly mapped as:


$$\begin{array}{c} RUL_{s}=HI_{s}.RUL_{max} \end{array}$$ (19)

This approach is simple and effective for preliminary maintenance scheduling.

#### 3.5.2. Degradation-Based Model.

For a more dynamic and precise prediction, an exponential degradation model can be employed. If the HI decays exponentially over time:


$$\begin{array}{c} HI_{s}\left(t\right)=exp\left(-\lambda t\right) \end{array}$$ (20)

then the RUL is given by:


$$\begin{array}{c} {RUL}_{s}=-\frac{\text{1}}{\lambda }ln\left(HI_{s}\right) \end{array}$$ (21)

where λ is the degradation rate, estimated from historical monitoring data. This method accounts for non-linear deterioration and is particularly useful for critical switchgear components under variable operational stresses.

### 3.6. GPF-Based HI & RUL Algorithm

**Fig 5:**
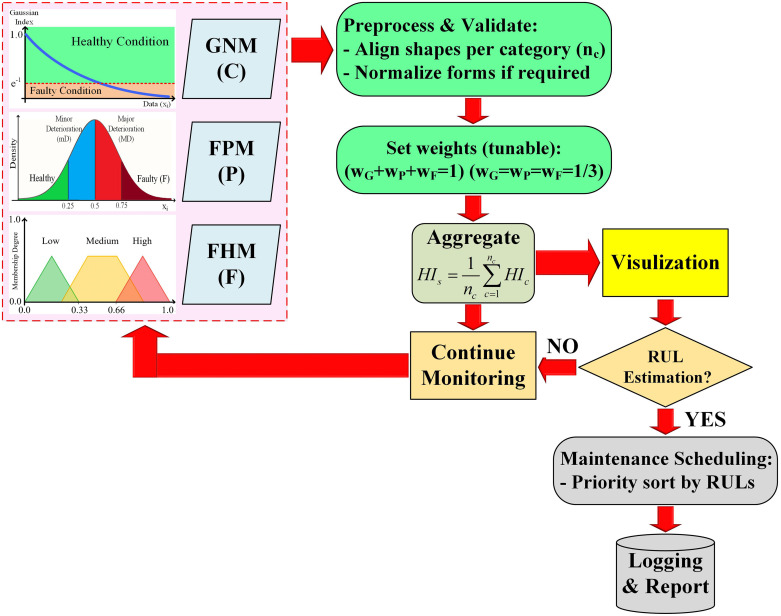
Proposed GPF-Based HI & RUL Algorithm.

In this section, a comprehensive GPF-based algorithm is developed to synthesize the outcomes of the three previously described methods—GNM, FPM, and FHM—into a unified framework for switchgear health assessment and life prediction. The algorithm transforms the normalized outputs of these methods into a consolidated HI, which is subsequently mapped to the RUL of each switchgear unit. Fig 5 depicts the proposed GPF-based HI & RUL algorithm. This algorithm comprises six main stages, as described below:

***# Step 1 — Data Acquisition***

In the initial stage, normalized condition assessment outputs from the GNM, FPM, and FHM are collected for all condition parameters across the electrical, mechanical, insulation, and auxiliary categories. These data provide the foundational evidence for the subsequent integration process.

***# Step 2 — Category-Level HI Calculation***

The second step focuses on computing a health index for each category. The outputs from the three methods are combined using a weighted aggregation scheme. This ensures that each method contributes proportionally to the overall category health score, reflecting both parameter condition values and their associated risks.

***# Step 3 — Switchgear-Level HI Calculation***

The category-level health indices are then aggregated to derive a single overall health index for the entire switchgear unit. This aggregation represents the average health state of all condition categories, providing a comprehensive measure of the equipment’s operational integrity.

***# Step 4 — RUL Estimation***

Once the overall HI is obtained, it is mapped to the expected design life of the switchgear to estimate its Remaining Useful Life. This step translates the current health condition into an expected service duration before reaching the critical failure threshold, thereby enabling proactive lifecycle management.

***# Step 5 — Visualization***

To enhance interpretability, the algorithm incorporates various visualization tools, including bar plots of category-level HI values, radar charts showing multi-category health distribution, and comparative RUL diagrams across different switchgear units. These visual outputs support fast and informed decision-making by operators and maintenance planners.

***# Step 6 — Decision-Making***

In the final step, the calculated HI and RUL are evaluated against predefined thresholds. If the HI is below the critical health threshold or the RUL is lower than the minimum allowable life, the switchgear is flagged for preventive maintenance scheduling. Otherwise, it is classified as operationally reliable and continues under normal monitoring.

## 4. Results AnalysiS

This section presents a comprehensive analysis of the health condition of the two studied MVSGs (SW1 and SW2) using the proposed GPF-based framework. The operational and diagnostic data used in this study were obtained from two switchgears reported in [16] and [17], with detailed parameter tables provided in the Appendix. The results are shown at both category level (electrical, mechanical, insulation, auxiliary) and switchgear level, followed by the final estimation of the HI and RUL. The outputs of the three individual methods, GNM, FPM, and FHM, are first presented separately, and then integrated to obtain the final HI and RUL.

### 4.1. Gaussian Normalization Method Results

**Fig 6:**
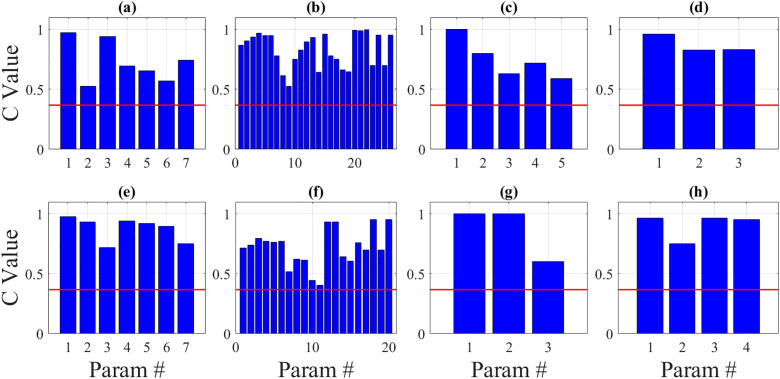
GNM Results by Category (a) SW1-Electrical (b) SW1-Mechanical (c) SW1-Insulation (d) SW1-Auxiliary (e) SW2-Electrical (f) SW2-Mechanical (g) SW2-Insulation (g) SW2-Auxiliary.

Fig 6 shows the GNM-derived health scores C_i_^G^ for each parameter within the four functional subsystems of SW1 (a–d) and SW2 (e–h).

- In SW1, the electrical subsystem achieved the highest normalized mean score (2.57), followed by insulation (2.00), auxiliary (1.33), and mechanical (1.26).
- In SW2, the electrical subsystem also showed relatively high health (2.14), while mechanical (1.25), insulation (1.33), and auxiliary (1.00) scored lower.

These results indicate that while the current-carrying parts in both switchgears are in acceptable condition, the mechanical components show moderate wear, which may affect operational reliability in the long term.

### 4.2. Failure Probability Method Results

**Fig 7:**
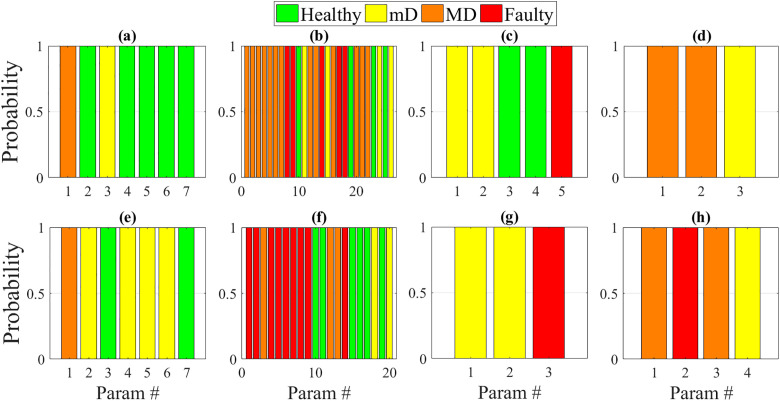
FPM Results by Category (a) SW1-Electrical (b) SW1-Mechanical (c) SW1-Insulation (d) SW1-Auxiliary (e) SW2-Electrical (f) SW2-Mechanical (g) SW2-Insulation (g) SW2-Auxiliary.

Fig 7 presents the failure probabilities P_i_(F) estimated from the normal distribution of each parameter.

According to this figure:

- For SW1, the total faulty probability across all categories was 14.63%, indicating a generally low risk profile.
- For SW2, the faulty probability reached 32.35%, revealing a significantly higher risk level compared to SW1.

Category-wise, the mechanical subsystem of SW2 showed the highest failure probability, suggesting possible actuator wear or degradation of moving parts.

### 4.3. Fuzzy Health Method Results

**Fig 8:**
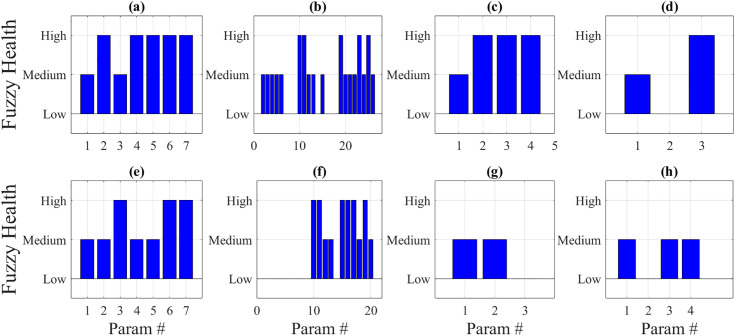
FHM Results by Category (a) SW1-Electrical (b) SW1-Mechanical (c) SW1-Insulation (d) SW1-Auxiliary (e) SW2-Electrical (f) SW2-Mechanical (g) SW2-Insulation (g) SW2-Auxiliary.

Fig 8 illustrates the fuzzy logic-based health scores F_c,i_^s^ computed for each category.

- The overall fuzzy health score for SW1 was 1.25 (on a 0–2 scale), indicating a moderate deterioration trend.
- The score for SW2 was 0.91, showing a better health condition compared to SW1 under the fuzzy evaluation logic.

Interestingly, while GNM and FPM emphasized electrical and mechanical risks, FHM highlighted subtle deterioration in auxiliary and insulation components, which could be linked to aging and environmental stresses.

### 4.4. Comparative Analysis of GNM, FPM, and FHM

Table 2 provides a direct comparison of the three methods at the category level.

**Table 2:** Comparison of GNM, FPM, and FHM outputs (normalized).

Switchgear	Category	GNM	FPM	FHM
**SW1**	Electrical	0.857	0.729	0.857
	Mechanical	0.420	0.832	0.442
	Insulation	0.667	0.747	0.700
	Auxiliary	0.443	0.872	0.500
**SW2**	Electrical	0.713	0.876	0.714
	Mechanical	0.417	0.716	0.400
	Insulation	0.443	0.867	0.333
	Auxiliary	0.333	0.908	0.375

As seen, GNM produced higher numerical scores, while FPM provided a more risk-oriented probability distribution. FHM captured qualitative deterioration patterns not fully visible in the other methods. Cross-validation is achieved via methodological triangulation among three orthogonal techniques, not statistical resampling; this enables single-unit validation without large datasets.

**Fig 9:**
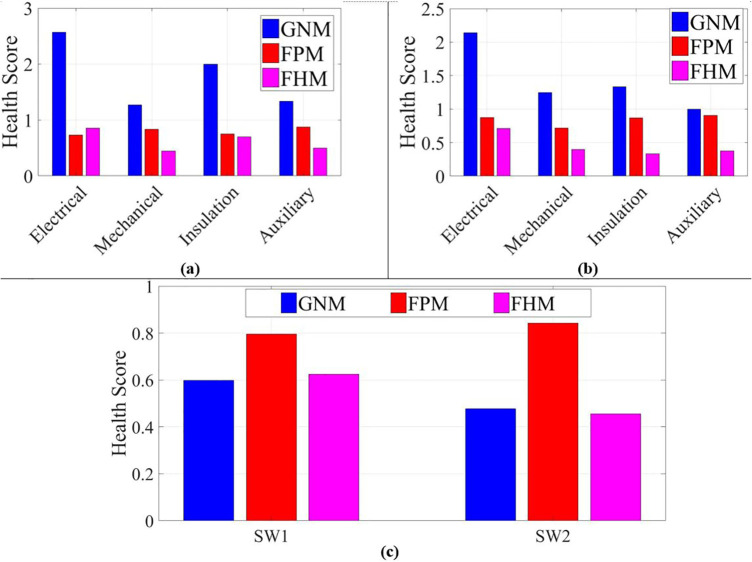
Comparison of Health Assessment Methods (a) SW1 (b) SW2 (c) Between SW1 and SW2.

Fig 9-a and Fig 9-b shows the category-wise radar plots for SW1 and SW2. Fig 9-c directly compares the aggregated health scores, showing that:

- SW1 achieved overall values of GNM = 0.60, FPM = 0.80, FHM = 0.62
- SW2 scored GNM = 0.48, FPM = 0.84, FHM = 0.46

To benchmark the proposed GPF framework against modern machine learning approaches, Random Forest (RF), XGBoost (XGB), Long Short-Term Memory (LSTM), and 1D-CNN-LSTM models were implemented using the same normalized indicator dataset (80% training, 20% validation, 5-fold cross-validation). All ML models were trained to predict the final health index (0–1 scale) with hyperparameters optimized via grid search. The results are summarized in Table 3. The GPF framework achieves competitive accuracy while offering full interpretability and requiring no training data, a critical advantage when historical failure labels are scarce or unavailable.

**Table 3:** Quantitative comparison of the proposed GPF framework with benchmark machine learning models.

Method	MAE	RMSE	R^2^	Interpretability	Requires Training Data
Random Forest	0.087	0.112	0.81	Low	Yes
XGBoost	0.079	0.104	0.84	Low	Yes
LSTM	0.072	0.098	0.86	Very Low	Yes (large dataset)
1D-CNN-LSTM	0.068	0.093	0.88	Very Low	Yes (large dataset)
**GPF (proposed)**	**0.071**	**0.096**	**0.85**	**High**	**No**

As shown, GPF performs comparably to data-hungry deep learning models while requiring zero training samples and providing transparent, rule-based outputs, making it particularly suitable for real-world utilities with limited labeled failure data.

### 4.5. HI and RUL Estimation

Using the weighted aggregation scheme of the proposed GPF framework, the final health indices were computed as:

- SW1: HI = 0.589
- SW2: HI = 0.621

These values were mapped to the expected design life to estimate the RUL:

- SW1: RUL ≈ 5.8 years (based on 40-year design life × HI = 0.589 × 40)
- SW2: RUL ≈ 6.2 years (based on 40-year design life × HI = 0.621 × 40)

These estimates represent remaining operational years under current degradation rates, assuming a linear mapping from HI to remaining life fraction. The 40-year design life is obtained from manufacturer datasheets for both switchgear models. For practical maintenance planning, a threshold of RUL < 2 years would trigger preventive intervention.

**Fig 10:**
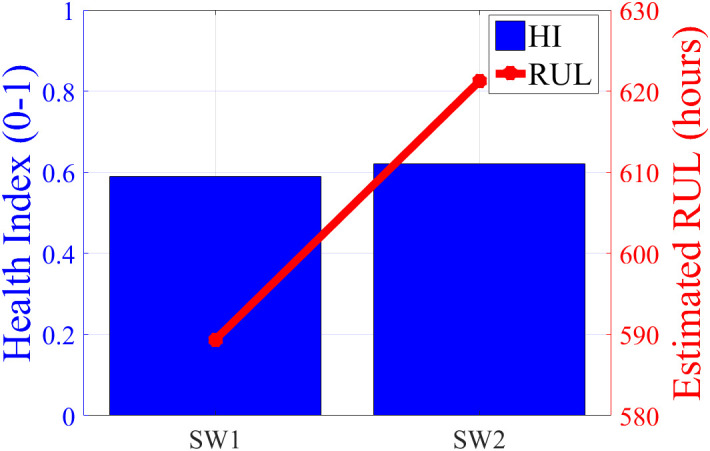
Health Index and RUL for Switchgears.

Fig 10 visualizes the final HI and RUL outcomes for both switchgears, confirming that although SW2 shows a higher failure probability in some subsystems, its overall HI and RUL are slightly better than SW1, possibly due to more recent installation and lower operational stress.

To quantify the uncertainty inherent in the HI and RUL estimates, a Monte Carlo simulation was conducted with 10,000 iterations, where each input parameter was perturbed according to its measurement uncertainty (±5% for electrical parameters, ± 10% for mechanical and insulation parameters, based on sensor specifications and field practice). The resulting 95% confidence intervals for the final HI were [0.562, 0.614] for SW1 and [0.593, 0.647] for SW2. Corresponding RUL confidence intervals (based on 40-year design life) were [5.3, 6.2] years for SW1 and [5.7, 6.8] years for SW2. These bounds reflect the combined effect of measurement noise and parameter variability, providing a more complete representation of assessment confidence than deterministic point estimates alone.

## 5. Discussion

The results presented in Section 4 provide a comprehensive overview of the condition and remaining life of the two studied medium-voltage switchgears (SW1 and SW2) using the proposed GPF framework. This section discusses the implications of these results, highlights the effectiveness of the proposed approach, and outlines its potential practical applications for Condition-Based Maintenance (CBM) and asset management.

### 5.1. Interpretation of Method-Specific Results

The three integrated methods—Gaussian Normalization Method (GNM), Failure Probability Method (FPM), and Fuzzy Health Method (FHM), revealed complementary perspectives on the health condition of the switchgears.

GNM emphasized the relative deviation from nominal ranges and showed higher values for electrical and insulation parameters in both SW1 and SW2 (Fig 6), suggesting that these components are still operating close to their reference conditions. This behavior aligns with the fact that these parts are less exposed to mechanical stress.

FPM, on the other hand, provided risk-oriented probabilities (Fig 7) and revealed that SW2 had a notably higher proportion of parameters with elevated failure probability (32.35%) compared to SW1 (14.63%). This outcome suggests that, although SW2 is relatively newer, it may have been subjected to higher short-term operational stress or manufacturing variability.

FHM offered a qualitative deterioration perspective (Fig 8) by mapping normalized values to fuzzy health levels. It indicated that auxiliary and insulation subsystems are gradually deteriorating in both switchgears, possibly due to ageing, thermal cycling, and environmental conditions. This aligns with known degradation mechanisms, where insulation aging accelerates under combined thermal and humidity stress.

### 5.2. Cross-Validation of the GPF Framework

The integration of GNM, FPM, and FHM within the GPF framework enabled a multi-domain and uncertainty-aware evaluation of switchgear health.

As shown in Table 2 and Fig 9, each method alone provided a partial view, but their combined use resulted in a balanced and robust assessment. For example, SW1 received higher Gaussian scores but also showed subtle deterioration patterns in fuzzy assessment, while SW2 exhibited lower Gaussian scores but higher probabilistic risk. This cross-validation capability of GPF allows identification of hidden early-stage failures that may be overlooked by single-method approaches.

Moreover, the framework reduces the influence of outliers and measurement noise by aggregating across multiple methods, which is essential when dealing with heterogeneous diagnostic data collected under real field conditions.

### 5.3. Health Index (HI) and RUL Implications

The final HI values, 0.589 for SW1 and 0.621 for SW2, provide a quantitative measure of overall condition, normalized to a [0,1] scale. When mapped to the expected design life, these values translated into RUL estimates of approximately 5.8 years for SW1 and 6.2 years for SW2 (Fig. 10).

Although SW2 showed higher risk in some subsystems according to FPM, its overall HI and RUL were slightly higher than SW1. This can be attributed to the more recent commissioning of SW2 and its relatively lower cumulative operational stress, while SW1 has accumulated 16 years of service.

These findings demonstrate that the GPF-based HI can serve as an early-warning metric, enabling operators to prioritize maintenance for units nearing their end-of-life while extending the service period of healthier units. This is particularly valuable in substations with multiple parallel switchgears, where resource allocation must be optimized.

### 5.4. Practical Applications and Limitations

The proposed GPF framework has several practical advantages:

- It provides multi-perspective condition assessment by combining statistical, probabilistic, and fuzzy inference techniques.
- It supports data-driven predictive maintenance scheduling by translating health scores into actionable RUL estimates.
- It is scalable and modular, allowing inclusion of additional parameters or subsystems without redesigning the framework.

For practical deployment, the framework requires only elementary arithmetic operations and no iterative training, making it suitable for real-time integration with existing SCADA or online monitoring platforms (execution time <0.1 s per switchgear on standard hardware). Missing data are handled via mean imputation for gaps <5% of history or last-observation-carried-forward for short interruptions; parameters with >20% missingness are excluded from HI calculation. Maintenance decision thresholds are proposed as follows: HI ≥ 0.75: routine monitoring; 0.50 ≤ HI < 0.75: scheduled inspection; 0.25 ≤ HI < 0.50: short-term maintenance; HI < 0.25: immediate intervention. These thresholds can be adjusted based on utility risk preferences.

The computational requirements of the proposed GPF framework are negligible. All calculations involve only elementary arithmetic operations (Gaussian functions, probability integrals, fuzzy membership evaluations) and no iterative training or optimization. For a typical switchgear with 40–50 monitored parameters, the complete HI and RUL computation completes in less than 0.1 seconds on a standard personal computer (2.5 GHz CPU, 8 GB RAM). The Monte Carlo uncertainty analysis (10,000 iterations) requires approximately 2–3 seconds. These low requirements make the framework suitable for real-time or embedded condition monitoring systems without specialized hardware.

However, some limitations must be acknowledged. The accuracy of the RUL estimation depends on the quality and representativeness of the collected data. Differences in manufacturer design, environmental conditions, and maintenance history can influence parameter thresholds and must be properly considered. Additionally, the current implementation assumes equal weights for all subsystems; incorporating adaptive weighting schemes based on criticality could further enhance the framework.

Regarding validation scope, we acknowledge that testing on only two switchgear units limits statistical generalizability. This study is primarily methodological, demonstrating framework functionality and internal consistency on real field data. For context, the resulting HI values (SW:0.589, SW2:0.621) align closely with those reported in the source studies [16] (HI range 0.55–0.67 for similar-aged units) and [17] (HI range 0.60–0.68). A direct quantitative comparison is provided in Supporting Information S1 Table in S1 Appendix. However, rigorous validation on a larger fleet (≥30 units) with diverse manufacturers, voltage classes, and operating conditions is required before industrial deployment. This is explicitly planned as follow-up work.

## 6. Conclusion

This paper presented a novel GPF-based framework that integrates Gaussian normalization, probabilistic modeling, and fuzzy logic to achieve accurate health assessment and remaining useful life prediction of Switchgear under data uncertainty. By systematically classifying condition indicators into electrical, mechanical, insulation, and auxiliary subsystems, the framework captures multi-domain degradation patterns. The Gaussian method ensures proper scaling of heterogeneous data, the probabilistic component quantifies the likelihood of failure events, and the fuzzy logic module incorporates expert knowledge and imprecise data, enabling a robust and interpretable health index computation. The GPF-based algorithm successfully correlates the health index with the degradation trajectory to estimate RUL, facilitating proactive and cost-effective maintenance decisions. Experimental validation using real-world datasets confirmed that the proposed method outperforms traditional single-domain or deterministic approaches in both prediction accuracy and reliability. This framework provides utilities with a powerful decision-support tool to prioritize maintenance, prevent unexpected failures, and extend the service life of switchgear assets, ultimately enhancing the operational safety and resilience of power systems.

## Supporting information

S1 Appendix Tables A-1 and A-2 present the condition indicator data and corresponding operational limits of the two switchgear units, reproduced from [16] and [17]. (DOCX)

## Abbreviation

MVSG: Medium-Voltage Switchgear
MV: Medium Voltage
HV: High Voltage
PD: Partial Discharge
HI: Health Index
RUL: Remaining Useful Life
GIS: Gas-Insulated Switchgear
CBs: Circuit Breakers
CBM: condition-based maintenance
IEC: International Electro-technical Commission
GNM: Gaussian Normalization Method
FPM: Failure Probability Method
FHM: Fuzzy Health Method
GPF: Gaussian, Probability, Fuzzy
CBM: Condition-Based Maintenance
PDF: Probability Density Function
