# Supplementary material for: An integrated Gaussian–Probabilistic–Fuzzy framework for health assessment and remaining useful life prediction of medium-voltage switchgears
Source: PLoS One. 2026 Jul 29;21(7):e0354713. doi: 10.1371/journal.pone.0354713 (PMC13419190; doi:10.1371/journal.pone.0354713)
Supplement: S1 Appendix — (DOCX) [file pone.0354713.s001.docx]

**APPENDIX**

**Tables A-1 and A-2 present the condition indicator data and corresponding operational limits of the two switchgear units, reproduced from [16] and [17].**

| **Table A-1.** SW 1 Condition Indicators Data [16]   \| **No.** \| **Indicators** \| **Value** \| **Limit** \| \| --- \| --- \| --- \| --- \| \| ***Electrical Subsystem*** \| \| \| \| \| E1.1 \| Cumulative s-c current (kA^2^) \| 6238 \| ≤15000 \| \| E1.2 \| Interruption number \| 18 \| ≤20 \| \| E1.3 \| S-c withstand current (kA) \| 25 \| pass \| \| E2.1 \| Contact resistance (μΩ) \| 40.1 \| ≤50 \| \| 41.3 \| ≤50 \| \| 43.7 \| ≤50 \| \| E2.2 \| Contact temperature (^o^C) \| 46.3 \| 60 \| \| ***Mechanical Subsystem*** \| \| \| \| \| M1.1 \| Close time (ms) \| 39.4 \| [30, 60] \| \| 40.3 \| [30, 60] \| \| 41.2 \| [30, 60] \| \| M1.1 \| Open time (ms) \| 21.2 \| [15, 30] \| \| 20.8 \| [15, 30] \| \| 20.8 \| [15, 30] \| \| M1.2 \| Close time difference (ms) \| 0.5 \| <2 \| \| Open time difference (ms) \| 0.3 \| <2 \| \| M1.3 \| Reclosing time (ms) \| 0.2 \| <2 \| \| M1.3 \| Close speed (m/s) \| 0.46 \| <0.6 \| \| M1.3 \| Open speed (m/s) \| 0.86 \| <1.2 \| \| M1.3 \| Contact bouncing time (ms) \| 1 \| <3 \| \| 1.1 \| <3 \| \| 0.5 \| <3 \| \| M2.2 \| Contact range (mm) \| 12 \| <3 \| \| M2.3 \| Contact overrun (mm) \| 5 \| <3 \| \| M2.3 \| Contact wear (mm) \| 0.7 \| <3 \| \| M4.1 \| Coil current signatures (ms) \| 0.45 \| [0,2.5] \| \| 9.07 \| [4.5,1 0] \| \| 14.78 \| [12, 18] \| \| M4.2 \| Contact voltage signatures (ms) \| 39.8 \| [38, 42] \| \| 39.9 \| [38, 42] \| \| M4.3 \| Trip coil resistance (μΩ) \| 200 \| >100 \| \| M4.3 \| close coil resistance (μΩ) \| 122.2 \| <200 \| \| M4.3 \| Trip coil resistance (insulation/DC) (μΩ) \| 200 \| >100 \| \| M4.3 \| close coil resistance (insulation/DC) (μΩ) \| 121.6 \| <200 \| \| ***Insulation Subsystem*** \| \| \| \| \| I1.1 \| Insulation resistance (MΩ) \| 5000 \| >1000 \| \| I2.1 \| Partial discharge (pC) \| 73.69 \| <100 \| \| I3.1 \| 1min withstand voltage (kV) \| 42 \| pass \| \| I3.1 \| Peak withstand voltage (kV) \| 63 \| pass \| \| I3.2 \| Ionic current (A) \| 68×10^-7 \| <5×10^-7 \| \| ***Auxiliary Subsystem*** \| \| \| \| \| A1.1 \| Service age (year/month) \| 15/11 \| <40 \| \| A2.1 \| Temperature (^o^C) \| 12.7 \| <45 \| \| A2.1 \| Humidity (%) \| 68 \| <95 \| | **Table A-2.** SW2 Condition Indicators Data [17]   \| **No.** \| **Indicators** \| **Value** \| **Limit** \| \| --- \| --- \| --- \| --- \| \| ***Electrical Subsystem*** \| \| \| \| \| E1.1 \| Cumulative s-c current (kA^2^) \| 8438 \| ≤20000 \| \| E1.2 \| Interruption number \| 19 \| ≤30 \| \| E1.3 \| S-c withstand current (kA) \| 31.5 \| pass \| \| E2.1 \| Contact resistance (μΩ) \| 28 \| ≤45 \| \| 29 \| ≤45 \| \| 30 \| ≤45 \| \| E2.2 \| Contact temperature (^o^C) \| 46 \| 60 \| \| ***Mechanical Subsystem*** \| \| \| \| \| M1.1 \| Close time (ms) \| 49.5 \| [40, 85] \| \| 50.1 \| [40, 85] \| \| 51.7 \| [40, 85] \| \| M1.1 \| Open time (ms) \| 31.1 \| [20, 65] \| \| 30.8 \| [20, 65] \| \| 31 \| [20, 65] \| \| M1.2 \| Close time difference (ms) \| 0.19 \| <2 \| \| Open time difference (ms) \| 0.31 \| <2 \| \| M1.3 \| Reclosing time (ms) \| 0.3 \| <2 \| \| M1.3 \| Close speed (m/s) \| 0.57 \| <0.6 \| \| M1.3 \| Open speed (m/s) \| 1.17 \| <1.2 \| \| M1.3 \| Contact bouncing time (ms) \| 1.1 \| <3 \| \| 1.1 \| <3 \| \| 0.5 \| <3 \| \| M2.1 \| Peak open coil current \| 6.83 \| < 8 \| \| M2.1 \| Peak close coil current \| 4.58 \| < 6 \| \| M2.2 \| Trip coil resistance (μΩ) \| 200 \| >100 \| \| M2.2 \| close coil resistance (μΩ) \| 122.2 \| <200 \| \| M2.2 \| Trip coil resistance (insulation/DC) (μΩ) \| 200 \| >100 \| \| M2.2 \| close coil resistance (insulation/DC) (μΩ) \| 121.6 \| <200 \| \| ***Insulation Subsystem*** \| \| \| \| \| I1.1 \| Insulation resistance (MΩ) \| 5000 \| >1000 \| \| I2.1 \| Partial discharge (dB) \| 4 \| <100 \| \| I3.2 \| Ionic current (A) \| 72×10^-7 \| pass \| \| ***Auxiliary Subsystem*** \| \| \| \| \| A1.1 \| Service age (year/month) \| 16/2 \|  \| \| A1.1 \| Contact wear (mm) \| 0.7 \| <3 \| \| A2.1 \| Temperature (^o^C) \| 18.2 \| <45 \| \| A2.1 \| Humidity (%) \| 58 \| <95 \| |
| --- | --- | --- | --- | --- | --- | --- | --- | --- | --- | --- | --- | --- | --- | --- | --- | --- | --- | --- | --- | --- | --- | --- | --- | --- | --- | --- | --- | --- | --- | --- | --- | --- | --- | --- | --- | --- | --- | --- | --- | --- | --- | --- | --- | --- | --- | --- | --- | --- | --- | --- | --- | --- | --- | --- | --- | --- | --- | --- | --- | --- | --- | --- | --- | --- | --- | --- | --- | --- | --- | --- | --- | --- | --- | --- | --- | --- | --- | --- | --- | --- | --- | --- | --- | --- | --- | --- | --- | --- | --- | --- | --- | --- | --- | --- | --- | --- | --- | --- | --- | --- | --- | --- | --- | --- | --- | --- | --- | --- | --- | --- | --- | --- | --- | --- | --- | --- | --- | --- | --- | --- | --- | --- | --- | --- | --- | --- | --- | --- | --- | --- | --- | --- | --- | --- | --- | --- | --- | --- | --- | --- | --- | --- | --- | --- | --- | --- | --- | --- | --- | --- | --- | --- | --- | --- | --- | --- | --- | --- | --- | --- | --- | --- | --- | --- | --- | --- | --- | --- | --- | --- | --- | --- | --- | --- | --- | --- | --- | --- | --- | --- | --- | --- | --- | --- | --- | --- | --- | --- | --- | --- | --- | --- | --- | --- | --- | --- | --- | --- | --- | --- | --- | --- | --- | --- | --- | --- | --- | --- | --- | --- | --- | --- | --- | --- | --- | --- | --- | --- | --- | --- | --- | --- | --- | --- | --- | --- | --- | --- | --- | --- | --- | --- | --- | --- | --- | --- | --- | --- | --- | --- | --- | --- | --- | --- | --- | --- | --- | --- | --- | --- | --- | --- | --- | --- | --- | --- | --- | --- | --- | --- | --- | --- | --- | --- | --- | --- | --- | --- | --- | --- | --- | --- | --- | --- | --- | --- | --- | --- | --- | --- | --- | --- | --- | --- | --- | --- | --- | --- | --- | --- | --- | --- | --- | --- | --- | --- | --- | --- | --- | --- | --- |
