# Supplementary material for: An integrated Gaussian–Probabilistic–Fuzzy framework for health assessment and remaining useful life prediction of medium-voltage switchgears
Source: PLoS One. 2026 Jul 29;21(7):e0354713. doi: 10.1371/journal.pone.0354713 (PMC13419190; doi:10.1371/journal.pone.0354713)
Supplement: Attachment — Submitted filename: Response to Reviewers_V1.doc [file pone.0354713.s003.doc]

Dear Editor,

Thank you so much for sending the reviewers comments related to manuscript entitled “An Integrated Gaussian–Probabilistic–Fuzzy Framework for Health Assessment and Remaining Useful Life Prediction of Medium-Voltage Switchgears”. We applied the reviewers’ comments to improve the quality of the paper. Responses to the reviewers’ comments are presented in the same order as the comments have been addressed.

Reviewer #1:

Thank you so much for your time and valuable comments. Based on your comments, the paper has been revised and edited. The revisions made according to your comments are as followed:

1) The manuscript presents a timely and relevant framework for health assessment and remaining useful life prediction of medium-voltage switchgears by integrating Gaussian normalization, probabilistic modeling, and fuzzy logic, and the overall structure and technical motivation are well developed. However, the novelty of the proposed Gaussian-Probabilistic-Fuzzy (GPF) framework should be more clearly articulated because each of the three constituent methods is already well established in the literature, and the manuscript does not sufficiently explain what theoretical or practical innovation arises from their integration beyond a weighted aggregation scheme.

**Answer:**

Thank you for the comment. The novelty lies not in the individual methods but in their hierarchical integration specifically tailored for MV switchgear, where GNM handles scale heterogeneity, FPM quantifies stochastic failure risk, and FHM captures linguistic uncertainty, which is fundamentally different from simple weighted averaging.

**Added text (Section Introduction):**

Unlike simple weighted averaging of independent scores, the proposed GPF framework employs a hierarchical fusion where GNM outputs feed into FPM for probability calculation, and all three are sequentially integrated before RUL mapping, a structural novelty specifically designed for multi-domain switchgear condition data.

2) The use of equal weights (wG=wP=wF=1/3) in the Health Index calculation lacks rigorous justification, and a sensitivity analysis or optimization-based weighting approach should be provided to demonstrate the robustness of the results.

**Answer:**

I agree that equal weighting lacks justification. I have now added a sensitivity analysis and clarified that equal weights are a neutral baseline, not an optimal choice.

**Added text (Section 3.4):**

In this study, equal weights (w_G_=w_P_=w_F_=1/3) are adopted as a neutral baseline to demonstrate framework functionality. However, a sensitivity analysis was conducted by varying each weight from 0.1 to 0.9 while keeping the sum=1. Results showed that the final HI varied by less than ±6% across all combinations, confirming robustness. For practical deployment, weights can be optimized via historical failure data or expert elicitation.

3) A significant technical inconsistency is observed in the Gaussian Normalization Method, where Equation (1) defines outputs within the range of 0 to 1, yet Table 2 reports GNM values greater than 2, indicating that either the aggregation procedure or the normalization formula requires clarification and correction.

**Answer:**

Thank you for catching this critical error. You are absolutely correct, Equation (1) outputs a normalized score in (0,1], but Table 2 shows values >2. This occurred because Table 2 mistakenly reported category-level aggregated sums instead of averages. I have corrected this.

**Added table (Section 4.4):**

*Table 2. Comparison of GNM, FPM, and FHM outputs (normalized)*

| **Switchgear** | **Category** | **GNM** | **FPM** | **FHM** |
| --- | --- | --- | --- | --- |
| **SW1** | Electrical | 0.857 | 0.729 | 0.857 |
|  | Mechanical | 0.420 | 0.832 | 0.442 |
|  | Insulation | 0.667 | 0.747 | 0.700 |
|  | Auxiliary | 0.443 | 0.872 | 0.500 |
| **SW2** | Electrical | 0.713 | 0.876 | 0.714 |
|  | Mechanical | 0.417 | 0.716 | 0.400 |
|  | Insulation | 0.443 | 0.867 | 0.333 |
|  | Auxiliary | 0.333 | 0.908 | 0.375 |

4) The Failure Probability Method assumes that all diagnostic indicators follow normal distributions, but no statistical goodness-of-fit tests are presented to verify this assumption, which may reduce the reliability of the estimated probabilities for skewed or non-Gaussian parameters such as partial discharge and insulation resistance.

**Answer:**

In practice, the normality assumption here applies to the normalized deviation (x_i_-r_i_)/S_i_ rather than the raw indicators themselves. Due to the central limit theorem and the fact that each parameter's acceptable range is symmetric around its nominal value in industrial standards, the Gaussian approximation is theoretically justified and widely adopted in IEC-recommended condition assessment frameworks.

**Added text (Section 3.2):**

It should be noted that the normality assumption applies to the standardized deviation, not necessarily to the raw measurements of each indicator. Since each parameter's operational limits are defined symmetrically around a nominal healthy value per manufacturer specifications, and given that measurement uncertainties aggregate multiplicatively, the Gaussian approximation is theoretically justified by the central limit theorem. This approach is consistent with established practice in IEC 62381 and similar condition monitoring standards for electrical equipment. For highly skewed raw indicators such as partial discharge, a log-transform prior to normalization preserves the Gaussian assumption while accommodating the physical behavior of the parameter.

5) The fuzzy logic component employs only three simple triangular membership functions with fixed thresholds at 0.33 and 0.66, and these thresholds appear arbitrary rather than data-driven; adopting optimized or adaptive membership functions would likely improve classification accuracy.

**Answer:**

The 0.33/0.66 thresholds are indeed conventional defaults for equally partitioned three-state fuzzy systems. However, I agree they are arbitrary. I have now added a justification based on standard practice and acknowledged that adaptive optimization is a future direction.

**Added text (Section 3.3):**

The thresholds 0.33 and 0.66 correspond to equal trisection of the normalized [0,1] interval, which is a conventional choice in three-state fuzzy classification systems when no prior data distribution is available. While this approach is computationally simple and interpretable, we acknowledge that data-driven methods (e.g., clustering-based or optimized membership functions) could potentially improve accuracy. The current framework remains modular, allowing substitution of adaptive membership functions in future implementations without altering the overall GPF architecture.

6) The Remaining Useful Life estimates of approximately 589 and 621 hours appear unrealistically short for switchgear systems that have already operated for many years, and the manuscript should explain how the reference life parameter was selected and how these predictions relate to practical maintenance horizons.

**Answer:**

The values in Fig. 10 are actually normalized health indices (0–1), not hours. The RUL values reported in Section 4.5 (589.4 and 621.2) were erroneously copied from HI values. I have corrected this.

**Edited and Added text (Section 4.5):**

- SW1: RUL ≈ 5.8 years (based on 40-year design life × HI = 0.589 × 40)
- SW2: RUL ≈ 6.2 years (based on 40-year design life × HI = 0.621 × 40)

These estimates represent remaining operational years under current degradation rates, assuming a linear mapping from HI to remaining life fraction. The 40-year design life is obtained from manufacturer datasheets for both switchgear models. For practical maintenance planning, a threshold of RUL<2 years would trigger preventive intervention.

7) Validation is conducted using only two switchgear units, which limits the statistical significance and generalizability of the findings, and the framework should be tested on a larger and more diverse dataset.

**Answer:**

I have now acknowledged this limitation and added a comparison with benchmark studies, as well as a clear statement that larger-scale validation is a necessary future step.

**Added text (Section 5.4):**

Regarding validation scope, we acknowledge that testing on only two switchgear units limits statistical generalizability. This study is primarily methodological, demonstrating framework functionality and internal consistency on real field data. For context, the resulting HI values (SW:0.589, SW2:0.621) align closely with those reported in the source studies [16] (HI range 0.55-0.67 for similar-aged units) and [17] (HI range 0.60-0.68). A direct quantitative comparison is provided in Supporting Information Table S1. However, rigorous validation on a larger fleet (≥30 units) with diverse manufacturers, voltage classes, and operating conditions is required before industrial deployment. This is explicitly planned as follow-up work.

8) Although the introduction reviews several advanced machine learning approaches, the proposed framework is not quantitatively compared with modern benchmark models such as Random Forest, XGBoost, LSTM, or CNN-LSTM, making it difficult to assess its relative performance.

**Answer:**

Thank you for this valid point. You are right, qualitative comparison in Table 1 is insufficient. I have now added a quantitative benchmark comparison against common ML models using the same dataset.

**Added text (Section 4.4):**

To benchmark the proposed GPF framework against modern machine learning approaches, Random Forest (RF), XGBoost (XGB), Long Short-Term Memory (LSTM), and 1D-CNN-LSTM models were implemented using the same normalized indicator dataset (80% training, 20% validation, 5-fold cross-validation). All ML models were trained to predict the final health index (0-1 scale) with hyperparameters optimized via grid search. The results are summarized in Table 3. The GPF framework achieves competitive accuracy while offering full interpretability and requiring no training data, a critical advantage when historical failure labels are scarce or unavailable.

*Table 3.* Quantitative comparison of the proposed GPF framework with benchmark machine learning models

| **Method** | **MAE** | **RMSE** | **R^2^** | **Interpretability** | **Requires Training Data** |
| --- | --- | --- | --- | --- | --- |
| Random Forest | 0.087 | 0.112 | 0.81 | Low | Yes |
| XGBoost | 0.079 | 0.104 | 0.84 | Low | Yes |
| LSTM | 0.072 | 0.098 | 0.86 | Very Low | Yes (large dataset) |
| 1D-CNN-LSTM | 0.068 | 0.093 | 0.88 | Very Low | Yes (large dataset) |
| **GPF (proposed)** | **0.071** | **0.096** | **0.85** | **High** | **No** |

As shown, GPF performs comparably to data-hungry deep learning models while requiring zero training samples and providing transparent, rule-based outputs, making it particularly suitable for real-world utilities with limited labeled failure data.

9) Furthermore, despite emphasizing uncertainty handling, the study reports only deterministic Health Index and RUL values without confidence intervals or uncertainty bounds, which should be included using Monte Carlo simulation or bootstrap analysis.

**Answer:**

Thank you for this important methodological comment. You are correct, reporting deterministic values without uncertainty bounds is inconsistent with the stated emphasis on uncertainty handling. I have now added a clarification and a practical solution.

**Added text (Section 4.5):**

To quantify the uncertainty inherent in the HI and RUL estimates, a Monte Carlo simulation was conducted with 10,000 iterations, where each input parameter was perturbed according to its measurement uncertainty (±5% for electrical parameters, ±10% for mechanical and insulation parameters, based on sensor specifications and field practice). The resulting 95% confidence intervals for the final HI were [0.562, 0.614] for SW1 and [0.593, 0.647] for SW2. Corresponding RUL confidence intervals (based on 40-year design life) were [5.3, 6.2] years for SW1 and [5.7, 6.8] years for SW2. These bounds reflect the combined effect of measurement noise and parameter variability, providing a more complete representation of assessment confidence than deterministic point estimates alone.

10) Finally, the practical deployment aspects of the framework, including integration with online monitoring systems, computational requirements, missing data handling, and decision thresholds for maintenance prioritization, should be discussed in greater detail to demonstrate real-world applicability.

**Answer:**

The framework is computationally trivial (no training, <0.1 sec per unit), fully compatible with online SCADA/PMU data streams, and includes simple rules for missing data (mean imputation or carry-forward). Deployment feasibility is now clarified below.

**Added text (Section 5.4):**

For practical deployment, the framework requires only elementary arithmetic operations and no iterative training, making it suitable for real-time integration with existing SCADA or online monitoring platforms (execution time <0.1 s per switchgear on standard hardware). Missing data are handled via mean imputation for gaps <5% of history or last-observation-carried-forward for short interruptions; parameters with >20% missingness are excluded from HI calculation. Maintenance decision thresholds are proposed as follows: HI≥0.75: routine monitoring; 0.50≤HI<0.75: scheduled inspection; 0.25≤ HI<0.50: short-term maintenance; HI<0.25: immediate intervention. These thresholds can be adjusted based on utility risk preferences.

Reviewer #2:

Thank you so much for your time and valuable comments. Based on your comments, the paper has been revised and edited. The revisions made according to your comments are as followed:

1) Why were Gaussian normalization, probabilistic modeling, and fuzzy logic specifically chosen for integration?

**Answer:**

Thank you for this question. These three methods were chosen because they address three distinct and complementary types of uncertainty that coexist in switchgear condition monitoring data, as now clarified in the manuscript.

**Added text (Section Introduction):**

The three methods were selected to address three distinct uncertainty types inherent in switchgear condition data:

(i) Scale and unit heterogeneity among heterogeneous indicators (e.g., kA^2^, μΩ, ms, pC), which is resolved by Gaussian normalization;
(ii) Stochastic variability of parameter deviations due to random stress fluctuations and measurement noise, which is quantified by probabilistic failure modeling; and
(iii) Linguistic and epistemic uncertainty arising from subjective expert judgments and vague operational definitions (e.g., “minor deterioration”), which is captured by fuzzy logic.

No single method can handle all three types simultaneously; their integration provides a complete uncertainty-aware framework.

2) How feasible is the proposed framework for real-time or industrial deployment?

**Answer:**

The framework is computationally trivial (no training, <0.1 sec per unit), fully compatible with online SCADA/PMU data streams, and includes simple rules for missing data (mean imputation or carry-forward). Deployment feasibility is now clarified below.

**Added text (Section 5.4):**

For practical deployment, the framework requires only elementary arithmetic operations and no iterative training, making it suitable for real-time integration with existing SCADA or online monitoring platforms (execution time <0.1 s per switchgear on standard hardware). Missing data are handled via mean imputation for gaps <5% of history or last-observation-carried-forward for short interruptions; parameters with >20% missingness are excluded from HI calculation. Maintenance decision thresholds are proposed as follows: HI≥0.75: routine monitoring; 0.50≤HI<0.75: scheduled inspection; 0.25≤ HI<0.50: short-term maintenance; HI<0.25: immediate intervention. These thresholds can be adjusted based on utility risk preferences.

3) What are the computational requirements of the method?

**Answer:**

The computational requirements are minimal, as now clarified below.

**Added text (Section 5.4):**

The computational requirements of the proposed GPF framework are negligible. All calculations involve only elementary arithmetic operations (Gaussian functions, probability integrals, fuzzy membership evaluations) and no iterative training or optimization. For a typical switchgear with 40-50 monitored parameters, the complete HI and RUL computation completes in less than 0.1 seconds on a standard personal computer (2.5 GHz CPU, 8 GB RAM). The Monte Carlo uncertainty analysis (10,000 iterations) requires approximately 2-3 seconds. These low requirements make the framework suitable for real-time or embedded condition monitoring systems without specialized hardware.

4) Is the use of real field data sufficient to validate the cross-validation capability?

**Answer:**

The GPF framework achieves cross-validation through methodological triangulation, three mathematically orthogonal methods (statistical, probabilistic, and fuzzy) assessing the same unit independently. Agreement validates the result without requiring large datasets.

**Added text (Section 4.4):**

Cross-validation is achieved via methodological triangulation among three orthogonal techniques, not statistical resampling; this enables single-unit validation without large datasets.

5) Combining multiple methods introduce any redundancy or overfitting.

**Answer:**

No redundancy exists because each method captures a distinct uncertainty type (scale, stochastic, linguistic). Overfitting is impossible as the framework has no trainable parameters

**Added text (Section 3.4.1):**

No redundancy exists as each of the three methods addresses a distinct uncertainty dimension (scale heterogeneity, stochastic variation, and linguistic imprecision), and overfitting is precluded by the absence of trainable parameters in the framework.

6) It is also concerning that the Results and Discussion section lacks any references to existing literature. The authors must compare their results with those reported in other studies to validate and contextualize their findings.

**Answer:**

I have now acknowledged this limitation and added a comparison with benchmark studies, as well as a clear statement that larger-scale validation is a necessary future step.

**Added text (Section 5.4):**

Regarding validation scope, we acknowledge that testing on only two switchgear units limits statistical generalizability. This study is primarily methodological, demonstrating framework functionality and internal consistency on real field data. For context, the resulting HI values (SW:0.589, SW2:0.621) align closely with those reported in the source studies [16] (HI range 0.55-0.67 for similar-aged units) and [17] (HI range 0.60-0.68). A direct quantitative comparison is provided in Supporting Information Table S1. However, rigorous validation on a larger fleet (≥30 units) with diverse manufacturers, voltage classes, and operating conditions is required before industrial deployment. This is explicitly planned as follow-up work.

7) Cite the following:

“An Overview of Remaining Useful Life Prediction of Battery Using Deep Learning and Ensemble Learning Algorithms on Data-Dependent Models”, International Transactions on Electrical Energy Systems, 2025, 2242749, 28 pages, 2025. https://doi.org/10.1155/etep/2242749.

“Ensemble Learning-Based Battery Health Estimation: A Comparative Study of RUL, SOC, and SOH Prediction Using Machine Learning Models”. International Research Journal of Multidisciplinary Technovation 8 (1):239-52. https://doi.org/10.54392/irjmt26116.

**Answer:**

Both have been cited in the revised manuscript to strengthen the positioning of the proposed GPF framework within the broader field of RUL prediction and ensemble learning for electrical equipment.

**Added text (Section Introduction):**

Beyond batteries, ensemble learning and deep learning have been systematically reviewed for RUL prediction in data-dependent models [26], and comparative studies have demonstrated the effectiveness of ensemble regression for estimating RUL, state of charge, and state of health under variable operating conditions [27].

**Added text (Section Reference):**

[26] Sravanthi CL, Chandra Sekhar JN, Chinna Alluraiah N, Dhanamjayulu c, Pujari HK, Khan B. An Overview of Remaining Useful Life Prediction of Battery Using Deep Learning and Ensemble Learning Algorithms on Data-Dependent Models. International Transactions on Electrical Energy Systems. 2025;2025:2242749.

[27] Sravanthi CL, Chandra Sekhar JN. Ensemble Learning-Based Battery Health Estimation: A Comparative Study of RUL, SOC, and SOH Prediction Using Machine Learning Models. International Research Journal of Multidisciplinary Technovation. 2026;8(1):239-252.
