# Supplementary material for: An integrated Gaussian–Probabilistic–Fuzzy framework for health assessment and remaining useful life prediction of medium-voltage switchgears
Source: PLoS One. 2026 Jul 29;21(7):e0354713. doi: 10.1371/journal.pone.0354713 (PMC13419190; doi:10.1371/journal.pone.0354713)
Supplement: Peer review history

# Peer review: Decision Letter 0

**DOI:** 10.1371/journal.pone.0354713.r001

**Authors:** J.N.Chandra Sekhar

13 May 2026

PONE-D-26-16090

An Integrated Gaussian–Probabilistic–Fuzzy Framework for Health Assessment and Remaining Useful Life Prediction of Medium-Voltage Switchgears

PLOS One

Dear Dr. Khorsandi,

Thank you for submitting your manuscript to PLOS ONE. After careful consideration, we feel that it has merit but does not fully meet PLOS ONE’s publication criteria as it currently stands. Therefore, we invite you to submit a revised version of the manuscript that addresses the points raised during the review process.

Please submit your revised manuscript by Jun 27 2026 11:59PM. If you will need more time than this to complete your revisions, please reply to this message or contact the journal office at plosone@plos.org. When you're ready to submit your revision, log on to https://www.editorialmanager.com/pone/ and select the 'Submissions Needing Revision' folder to locate your manuscript file.

Please include the following items when submitting your revised manuscript:

- A letter that responds to each point raised by the academic editor and reviewer(s). You should upload this letter as a separate file labeled 'Response to Reviewers'.
- A marked-up copy of your manuscript that highlights changes made to the original version. You should upload this as a separate file labeled 'Revised Manuscript with Track Changes'.
- An unmarked version of your revised paper without tracked changes. You should upload this as a separate file labeled 'Manuscript'.

If you would like to make changes to your financial disclosure, please include your updated statement in your cover letter. Guidelines for resubmitting your figure files are available below the reviewer comments at the end of this letter.

If applicable, we recommend that you deposit your laboratory protocols in protocols.io to enhance the reproducibility of your results. Protocols.io assigns your protocol its own identifier (DOI) so that it can be cited independently in the future. For instructions see: https://journals.plos.org/plosone/s/submission-guidelines#loc-laboratory-protocols. Additionally, PLOS ONE offers an option for publishing peer-reviewed Lab Protocol articles, which describe protocols hosted on protocols.io. Read more information on sharing protocols at https://plos.org/protocols?utm_medium=editorial-email&utm_source=authorletters&utm_campaign=protocols.

As the corresponding author, your ORCID iD is verified in the submission system and will appear in the published article. PLOS supports the use of ORCID, and we encourage all coauthors to register for an ORCID iD and use it as well. Please encourage your coauthors to verify their ORCID iD within the submission system before final acceptance, as unverified ORCID iDs will not appear in the published article. *Only*  the individual author can complete the verification step; PLOS staff *cannot*  verify ORCID iDs on behalf of authors.

We look forward to receiving your revised manuscript.

Kind regards,

J.N.Chandra Sekhar, Ph.D.

Academic Editor

PLOS One

**Journal Requirements:**

When submitting your revision, we need you to address these additional requirements.

1. Please ensure that your manuscript meets PLOS ONE's style requirements, including those for file naming. The PLOS ONE style templates can be found at

https://journals.plos.org/plosone/s/file?id=wjVg/PLOSOne_formatting_sample_main_body.pdf and

https://journals.plos.org/plosone/s/file?id=ba62/PLOSOne_formatting_sample_title_authors_affiliations.pdf

2. We note that your Data Availability Statement is currently as follows:

“All relevant data are within the manuscript and its Supporting Information files.”

Please confirm at this time whether or not your submission contains all raw data required to replicate the results of your study. Authors must share the “minimal data set” for their submission. PLOS defines the minimal data set to consist of the data required to replicate all study findings reported in the article, as well as related metadata and methods (https://journals.plos.org/plosone/s/data-availability#loc-minimal-data-set-definition).

For example, authors should submit the following data:

- The values behind the means, standard deviations and other measures reported;

- The values used to build graphs;

- The points extracted from images for analysis.

Authors do not need to submit their entire data set if only a portion of the data was used in the reported study.

If your submission does not contain these data, please either upload them as Supporting Information files or deposit them to a stable, public repository and provide us with the relevant URLs, DOIs, or accession numbers. For a list of recommended repositories, please see https://journals.plos.org/plosone/s/recommended-repositories.

If there are ethical or legal restrictions on sharing a de-identified data set, please explain them in detail (e.g., data contain potentially sensitive information, data are owned by a third-party organization, etc.) and who has imposed them (e.g., an ethics committee). Please also provide contact information for a data access committee, ethics committee, or other institutional body to which data requests may be sent. If data are owned by a third party, please indicate how others may request data access.

3. If the reviewer comments include a recommendation to cite specific previously published works, please review and evaluate these publications to determine whether they are relevant and should be cited. There is no requirement to cite these works unless the editor has indicated otherwise.

[Note: HTML markup is below. Please do not edit.]

Reviewers' comments:

Reviewer's Responses to Questions

**Comments to the Author**

1. Is the manuscript technically sound, and do the data support the conclusions?

The manuscript must describe a technically sound piece of scientific research with data that supports the conclusions. Experiments must have been conducted rigorously, with appropriate controls, replication, and sample sizes. The conclusions must be drawn appropriately based on the data presented.

Reviewer #1: Yes

Reviewer #2: Yes

**********

2. Has the statistical analysis been performed appropriately and rigorously?

Reviewer #1: Yes

Reviewer #2: Yes

**********

3. Have the authors made all data underlying the findings in their manuscript fully available?

The PLOS Data policy requires authors to make all data underlying the findings described in their manuscript fully available without restriction, with rare exception (please refer to the Data Availability Statement in the manuscript PDF file). The data should be provided as part of the manuscript or its supporting information, or deposited to a public repository. For example, in addition to summary statistics, the data points behind means, medians and variance measures should be available. If there are restrictions on publicly sharing data—e.g. participant privacy or use of data from a third party—those must be specified.

Reviewer #1: Yes

Reviewer #2: Yes

**********

4. Is the manuscript presented in an intelligible fashion and written in standard English?

PLOS ONE does not copyedit accepted manuscripts, so the language in submitted articles must be clear, correct, and unambiguous. Any typographical or grammatical errors should be corrected at revision, so please note any specific errors here.

Reviewer #1: Yes

Reviewer #2: Yes

**********

5. Review Comments to the Author

Please use the space provided to explain your answers to the questions above. You may also include additional comments for the author, including concerns about dual publication, research ethics, or publication ethics. (Please upload your review as an attachment if it exceeds 20,000 characters)

Reviewer #1: The manuscript presents a timely and relevant framework for health assessment and remaining useful life prediction of medium-voltage switchgears by integrating Gaussian normalization, probabilistic modeling, and fuzzy logic, and the overall structure and technical motivation are well developed. However, the novelty of the proposed Gaussian–Probabilistic–Fuzzy (GPF) framework should be more clearly articulated because each of the three constituent methods is already well established in the literature, and the manuscript does not sufficiently explain what theoretical or practical innovation arises from their integration beyond a weighted aggregation scheme. The use of equal weights (wG = wP = wF = 1/3) in the Health Index calculation lacks rigorous justification, and a sensitivity analysis or optimization-based weighting approach should be provided to demonstrate the robustness of the results. A significant technical inconsistency is observed in the Gaussian Normalization Method, where Equation (1) defines outputs within the range of 0 to 1, yet Table 2 reports GNM values greater than 2, indicating that either the aggregation procedure or the normalization formula requires clarification and correction. The Failure Probability Method assumes that all diagnostic indicators follow normal distributions, but no statistical goodness-of-fit tests are presented to verify this assumption, which may reduce the reliability of the estimated probabilities for skewed or non-Gaussian parameters such as partial discharge and insulation resistance. The fuzzy logic component employs only three simple triangular membership functions with fixed thresholds at 0.33 and 0.66, and these thresholds appear arbitrary rather than data-driven; adopting optimized or adaptive membership functions would likely improve classification accuracy. The Remaining Useful Life estimates of approximately 589 and 621 hours appear unrealistically short for switchgear systems that have already operated for many years, and the manuscript should explain how the reference life parameter was selected and how these predictions relate to practical maintenance horizons. Validation is conducted using only two switchgear units, which limits the statistical significance and generalizability of the findings, and the framework should be tested on a larger and more diverse dataset. Although the introduction reviews several advanced machine learning approaches, the proposed framework is not quantitatively compared with modern benchmark models such as Random Forest, XGBoost, LSTM, or CNN-LSTM, making it difficult to assess its relative performance. Furthermore, despite emphasizing uncertainty handling, the study reports only deterministic Health Index and RUL values without confidence intervals or uncertainty bounds, which should be included using Monte Carlo simulation or bootstrap analysis. Finally, the practical deployment aspects of the framework, including integration with online monitoring systems, computational requirements, missing data handling, and decision thresholds for maintenance prioritization, should be discussed in greater detail to demonstrate real-world applicability. Overall, the manuscript addresses an important problem and offers a promising methodology, but substantial revisions are needed to strengthen the theoretical rigor, numerical consistency, validation depth, and practical relevance of the proposed approach.

Reviewer #2: 1. Why were Gaussian normalization, probabilistic modeling, and fuzzy logic specifically chosen for integration?

2. How feasible is the proposed framework for real-time or industrial deployment?

3. What are the computational requirements of the method?

4. Is the use of real field data sufficient to validate the cross-validation capability?

5. Combining multiple methods introduce any redundancy or overfitting.

6. It is also concerning that the Results and Discussion section lacks any references to existing literature. The authors must compare their results with those reported in other studies to validate and contextualize their findings.

7. Cite the following

� “An Overview of Remaining Useful Life Prediction of Battery Using Deep Learning and Ensemble Learning Algorithms on Data-Dependent Models”, International Transactions on Electrical Energy Systems, 2025, 2242749, 28 pages, 2025. https://doi.org/10.1155/etep/2242749.

� “Ensemble Learning-Based Battery Health Estimation: A Comparative Study of RUL, SOC, and SOH Prediction Using Machine Learning Models”. International Research Journal of Multidisciplinary Technovation 8 (1):239-52. https://doi.org/10.54392/irjmt26116.

**********

6. PLOS authors have the option to publish the peer review history of their article (what does this mean?). If published, this will include your full peer review and any attached files.

If you choose “no”, your identity will remain anonymous but your review may still be made public.

**Do you want your identity to be public for this peer review?**  For information about this choice, including consent withdrawal, please see our Privacy Policy.

Reviewer #1: No

Reviewer #2: No

**********

[NOTE: If reviewer comments were submitted as an attachment file, they will be attached to this email and accessible via the submission site. Please log into your account, locate the manuscript record, and check for the action link "View Attachments". If this link does not appear, there are no attachment files.]

To ensure your figures meet our technical requirements, please review our figure guidelines: https://journals.plos.org/plosone/s/figures

You may also use PLOS’s free figure tool, NAAS, to help you prepare publication quality figures: https://journals.plos.org/plosone/s/figures#loc-tools-for-figure-preparation.

NAAS will assess whether your figures meet our technical requirements by comparing each figure against our figure specifications.

# Author comment: Author response to Decision Letter 1

**DOI:** 10.1371/journal.pone.0354713.r002

11 Jun 2026

Please refer to the response letter.

Attachment Submitted filename: Response to Reviewers_V1.doc

# Editor report: Decision Letter 1

**DOI:** 10.1371/journal.pone.0354713.r003

**Authors:** J.N.Chandra Sekhar

13 Jul 2026

An Integrated Gaussian–Probabilistic–Fuzzy Framework for Health Assessment and Remaining Useful Life Prediction of Medium-Voltage Switchgears

PONE-D-26-16090R1

Dear Dr. Amir Khorsandi

We’re pleased to inform you that your manuscript has been judged scientifically suitable for publication and will be formally accepted for publication once it meets all outstanding technical requirements.

Within one week, you’ll receive an e-mail detailing the required amendments. When these have been addressed, you’ll receive a formal acceptance letter and your manuscript will be scheduled for publication.

An invoice will be generated when your article is formally accepted. Please note, if your institution has a publishing partnership with PLOS and your article meets the relevant criteria, all or part of your publication costs will be covered. Please make sure your user information is up-to-date by logging into Editorial Manager at Editorial Manager® and clicking the ‘Update My Information' link at the top of the page. For questions related to billing, please contact billing support.

If your institution or institutions have a press office, please notify them about your upcoming paper to help maximize its impact. If they’ll be preparing press materials, please inform our press team as soon as possible -- no later than 48 hours after receiving the formal acceptance. Your manuscript will remain under strict press embargo until 2 pm Eastern Time on the date of publication. For more information, please contact onepress@plos.org.

Kind regards,

J.N.Chandra Sekhar, Ph.D.

Academic Editor

PLOS One

Additional Editor Comments (optional):

Reviewers' comments:

# Editor report: Acceptance letter

**DOI:** 10.1371/journal.pone.0354713.r004

**Authors:** J.N.Chandra Sekhar

PONE-D-26-16090R1

PLOS One

Dear Dr. Khorsandi,

I'm pleased to inform you that your manuscript has been deemed suitable for publication in PLOS One. Congratulations! Your manuscript is now being handed over to our production team.

At this stage, our production department will prepare your paper for publication. This includes ensuring the following:

* All references, tables, and figures are properly cited

* All relevant supporting information is included in the manuscript submission,

* There are no issues that prevent the paper from being properly typeset

You will receive further instructions from the production team, including instructions on how to review your proof when it is ready. Please keep in mind that we are working through a large volume of accepted articles, so please give us a few days to review your paper and let you know the next and final steps.

Lastly, if your institution or institutions have a press office, please let them know about your upcoming paper now to help maximize its impact. If they'll be preparing press materials, please inform our press team within the next 48 hours. Your manuscript will remain under strict press embargo until 2 pm Eastern Time on the date of publication. For more information, please contact onepress@plos.org.

You will receive an invoice from PLOS for your publication fee after your manuscript has reached the completed accept phase. If you receive an email requesting payment before acceptance or for any other service, this may be a phishing scheme. Learn how to identify phishing emails and protect your accounts at https://explore.plos.org/phishing.

If we can help with anything else, please email us at customercare@plos.org.

Thank you for submitting your work to PLOS ONE and supporting open access.

Kind regards,

PLOS ONE Editorial Office Staff

on behalf of

Dr. J.N.Chandra Sekhar

Academic Editor

PLOS One
